# The Role of Gut Microflora and the Cholinergic Anti-inflammatory Neuroendocrine System in Diabetes Mellitus

**DOI:** 10.3389/fendo.2016.00055

**Published:** 2016-06-08

**Authors:** Parth J. Parekh, Vipul R. Nayi, David A. Johnson, Aaron I. Vinik

**Affiliations:** ^1^Division of Gastroenterology and Hepatology, Department of Internal Medicine, Tulane University, New Orleans, LA, USA; ^2^Department of Internal Medicine, Montefiore Medical Center, Albert Einstein College of Medicine, Bronx, NY, USA; ^3^Division of Gastroenterology, Department of Internal Medicine, Eastern Virginia Medical School, Norfolk, VA, USA; ^4^Division of Endocrinology, Department of Internal Medicine, Eastern Virginia Medical School, Norfolk, VA, USA

**Keywords:** diabetes, intestinal microbiome, autonomic nervous system, intestinal dysbiosis, metabolic syndrome, obesity

## Abstract

The obesity epidemic has drastically impacted the state of health care in the United States. Paralleling this epidemic is the incidence of diabetes mellitus, with a notable shift toward a much younger age of onset. While central to the pathogenesis of diabetes associated with obesity is the role of inflammation attributed to “adiposopathy.” Emerging data suggest that changes in sympathetic/parasympathetic balance regulated by the brain precede changes in the inflammatory cascade. It has now been established that the gut microflora contributes significantly to the activation and inhibition of autonomic control and impact the set of the neuroinflammatory inhibitory reflex mediated by the cholinergic nervous system. There has been a paradigm shift toward further investigating commensal bacteria in the pathogenesis of obesity and diabetes mellitus and its complications, as dysbiosis is thought to play a pivotal role in diabetic-associated disorders. This paper is intended to evaluate the role of intestinal dysbiosis in the pathogenesis of diabetes mellitus and examine the potential for restoration of balance *via* use of probiotics.

## Introduction

The incidence of diabetes mellitus continues to rise at historic rates, paralleling that of the obesity epidemic (1). Superimposed on this worldwide epidemic, clinicians are seeing a demographic trend to an even younger age of diagnosis of diabetes mellitus type 2 (T2DM) (2). Currently, therapeutic approaches mainly target the sequelae of disease, whereas research efforts are directed toward addressing the underlying cause of impaired metabolism (3, 4). There has been considerable interest in the role of autonomic balance (5, 6) and the intestinal microbiota may play in this disease, with emerging data implicating a brain–gut dysbiosis in the pathogenesis of obesity, diabetes, and metabolic syndrome (7). In this review, we examine the most current literature focusing on the emerging links between the gut microbiome brain axis and its role in the pathogenesis of diabetes mellitus and its complications.

## “Metabolic Infection” – Microbiota and a Low-Grade Inflammatory State

The metabolic alterations associated with obesity also cause a chronic low-grade inflammatory state, which affects energy homeostasis and glucose metabolism (7). Diabetes mellitus has long been thought of as low-grade inflammatory state, secondary to adipocyte necrosis (8–10). The imbalance between caloric intake and energy expenditure results in adipocyte hypertrophy, culminating in local hypoxia and apoptosis (10). As a result, these adipocytes begin to secrete TNF-α in low quantities, thereby stimulating a chemotactic response (7, 10).

Cani et al. suggested that bacterial lipopolysaccharide (LPS) was responsible for this low-grade inflammatory state (11). They demonstrated that levels of endotoxemia fluctuated depending on whether or not the host was in a fasting or fed state. After a 4-week high-fat diet was introduced, levels of LPS-producing microbiota had significantly increased. In addition, continuous subcutaneous infusion of LPS resulted in fasting hyperglycemia and hyperinsulinemia, associated with whole-body, liver, and adipose tissue weight gain that was comparable to those mice fed a high-fat diet for 4 weeks. As the hepatic, but not whole-body, insulin resistance was seen in LPS-infused mice, the authors concluded that metabolic endotoxemia results in a low-grade inflammatory state and acts as a trigger for insulin resistance and the onset of diabetes and obesity. The findings by Cani et al. indicate that LPS is in fact a trigger for the early development of metabolic disease and is involved early in the inflammatory cascade as it stimulates a number of key cytokines (evidenced by increased expression of genes encoding IL-6, TNF-α, IL-1, and PAI-1 in adipose, liver, and muscle). This upregulation is pivotal in the development of insulin resistance *via* its effect on mCD14 and toll-like receptor (TLR)-4.

Remely et al. evaluated the impact of intestinal dysbiosis on the upregulation of pro-inflammatory cytokines, namely, TLRs 2 and 4 in three groups of subjects: subjects with T2DM receiving glucagon-like peptide-1 (GLP-1) agonist therapy, obese subjects without established insulin resistance, and a lean normative control group (12). They found there to be a significantly higher ratio of *Firmicutes*:*Bacteroides* in the diabetes cohort compared to their lean and obese counterparts. In addition, *Faecalibacterium prausnitzii* were least abundant in the diabetes cohort and most prevalent in lean controls. Methylation analysis demonstrated significantly lower methylation of TLRs 2 and 4 in the diabetic group compared to obese and lean controls, with levels significantly correlating with body mass index. The authors suggested that the changes in commensal microbiota-induced changes and subsequent alterations in cell components are involved in the epigenetic regulation of the inflammatory cascade. Endotoxin LPS is invariably associated with Gram-negative bacteria whether the organisms pathogenic or not. While normally LPS endotoxemia triggers the inflammatory process by binding to the TLR-4 complex at the surface of innate immune cells triggering the host inflammatory cascade, ultimately resulting in insulin resistance; counterintuitively, it was lower levels of Gram-negative *Bacteroides* that was associated with diabetes. One possible explanation is the impaired innate immune system. The commensal relationship between host and the gut microbiome is pivotal in maintaining the integrity of the immune system. *Bacteroides* has been demonstrated to influence the activity of defensins (e.g., angiogenin), which regulated the microbial system and innate immunity when secreted; thus, a populous deficient would be predisposed to intestinal dysbiosis and inflammation (13).

Apelin is hormone produced by adipose tissue that is thought to play a key role in the regulation of homeostasis and low-grade inflammation (14, 15). Geurts et al. investigated the microbial composition in obese and diabetic leptin-resistant mice (*db/db*) in order to establish the role of specific microbial strains and their gut-derived compounds, i.e., LPS, in adipose tissue metabolism *via* the endocannabinoid system (14). They found *db/db* mice to have a significantly higher populous of *Firmicutes*, *Proteobacteria*, and *Fibrobacteres* phyla compared to the lean cohort. In addition, they were able to demonstrate the roles of the endocannabinoid system and LPS in regulating apelinergic tone. Using *in vitro* and *in vivo* models, they found both the endocannabinoid system and low-grade inflammation to regulate apelin expression in adipose tissue. Lastly, microbiota profiling revealed the commensal microbiota of T2DM mice to significantly differ from that of their lean counterparts, indicating a specific relationship between the microbiome and regulation of the apelinergic system.

The next step was to demonstrate the role of intestinal dysbiosis in the occurrence of diabetes mellitus. Cani et al. administered antibiotics in order to alter commensal microbiota to evaluate whether intestinal dysbiosis is responsible for metabolic endotoxemia, low-grade inflammation, obesity, and diabetes and also to determine what underlying mechanisms are at play (16). They found that antibiotic-induced microbial changes had reduced metabolic endotoxemia and cecal content of LPS compared to high-fat fed mice and genetically obese mice (*ob*/*ob*). This correlated with reduced glucose tolerance, inflammation, oxidative stress, body weight gain, development of fat mass, and macrophage infiltration marker mRNA expression in visceral adipose tissue. Mice subjected to high-fat feeding strongly increased intestinal permeability and reduced expression of proteins responsible for tight junctions. Lastly, genetically altered mice that lack functional LPS receptors (CD14 knockout mice) exhibited similar characteristics as mice subjected to antibiotics (i.e., resistant to diet-induced obesity and hepatic insulin resistance). Subsequent metagenome-wide studies have demonstrated the presence of intestinal dysbiosis in patients with T2DM (17, 18), of interest a decrease in the abundance of some universal butyrate-producing bacteria (17), which can alter energy homeostasis, further supporting the concept of intestinal dysbiosis and metabolic endotoxemia and its sequelae set forth by Cani et al. (16).

More recent studies have aimed to investigate the impact of diet on alteration of commensal microbiota and potentially identify specific bacterial strains that predominate diabetes-induced low-grade inflammation. Fallucca et al. investigated the use of the macrobiotic Ma-Pi 2 diet in patients with T2DM for 21 days (19). The Ma-Pi2 diet is one that is low in fat, protein, and fructose containing no animal protein or sugar. It is rich in complex carbohydrates, natural fibers, and most significantly prebiotic and probiotic products (20). The investigators found there to be significant reduction of fasting blood glucose, plasma lipid fractions, plasma insulin, and energy homeostasis by implementing the Ma-Pi 2 diet supporting the role of intestinal dysbiosis in the development of diabetes mellitus and that manipulation of the gut microbiome may be a promising therapeutic option. Subsequent studies have also demonstrated the Ma-Pi 2 diet to reduce markers of insulin resistance, inflammation, and insulin growth factor-1, supporting its use in patients with T2DM (21–23). None of these studies aimed to understand the link between the intestinal dysbiosis and the metabolic derangements found.

## Role of Specific Bacterial Strains

**Figure 1:**
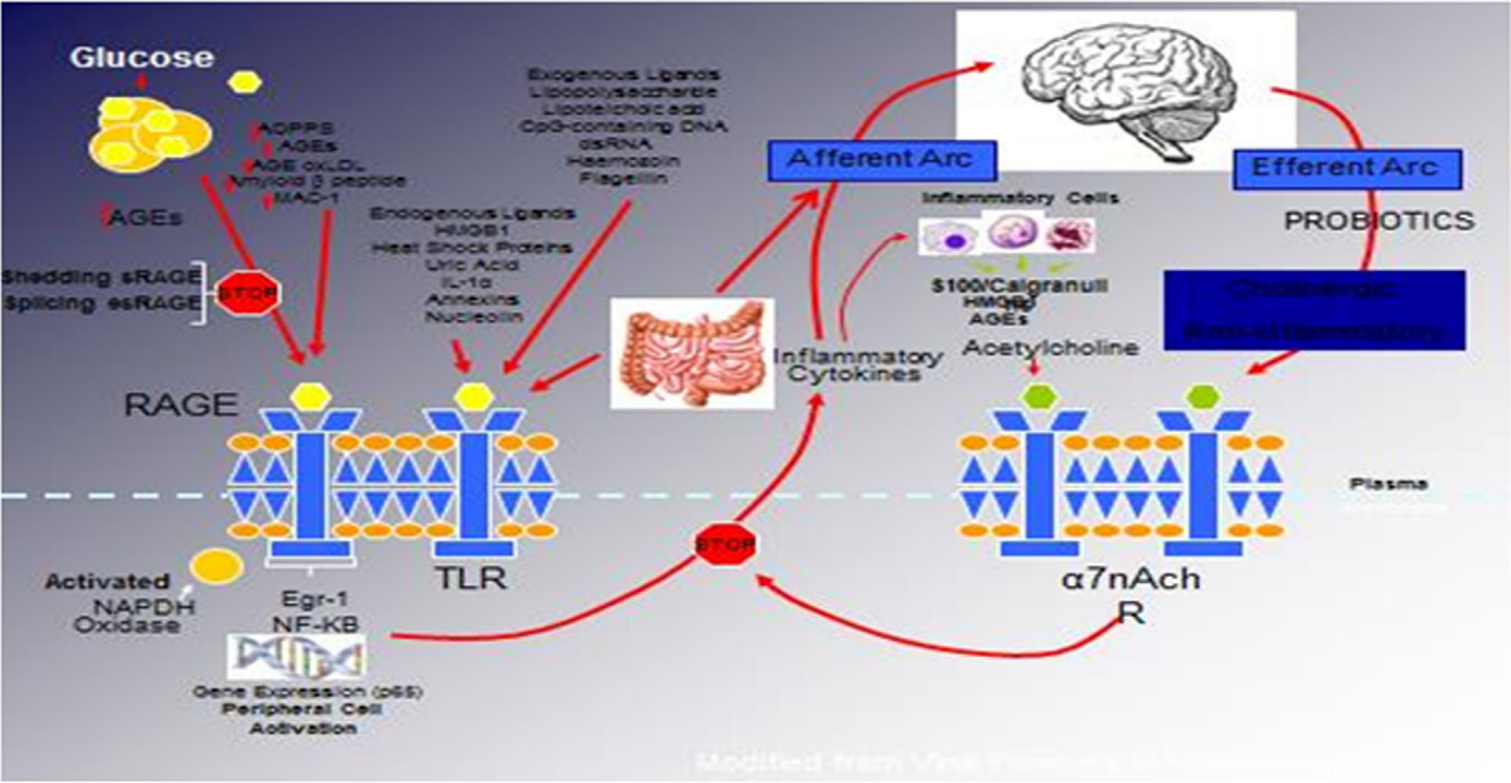
**The relationship between binding of ligands to the pattern recognition AGE receptor (RAGE) and inflammation, gene expression, oxidative and nitrosative stress, and activation of an inflammatory cascade involving a cadre of inflammatory cytokines as an afferent arc impacting the brainstem nuclei initiating an efferent arc, in which acetylcholine binds to its receptor inhibiting the activation of the inflammatory cascade**.

Several studies have suggested the protective benefit of *Lactobacillus casei* against diabetes mellitus (24–29). A recent study by Chen et al. evaluated the antidiabetic effects of *L. casei* on mice using a model of T2DM induced by a high-fat diet and low-dose streptozotocin (24). Administration of *L. casei* significantly reduced fasting and postprandial 2-h serum glucose levels, hemoglobin A1c levels, other markers of metabolic syndrome [e.g., triglycerides, total cholesterol, and low-density lipoprotein cholesterol (LDL-C)], and markers of inflammation (e.g., TNF-α, endotoxin) compared to control. In addition, islets of Langerhans were substantially protected against destruction when exposed to *L. casei* compared to controls, which led to the conclusion that oral administration of *L. casei* may have a role in the primary prevention of diabetes mellitus. A subsequent study by Zhang et al. postulated that *L. casei* might induce its antidiabetic effects by acting on the bile acid-chloride transport (25), while we will suggest an alternative mechanism as detailed in the enhancement of the neuroinflammatory reflex (see Figure 1).

Unequivocal evidence demonstrates the impact of the gut microbiota on whole-body metabolism and energy homeostasis (7); however, the microbial composition and the underlying mechanisms of interaction between host and commensal microbiota that impact host–gut barrier function and metabolism remain unclear. Recent efforts have shifted focus onto the potential role *Akkermansia muciniphila* (30–37) and *F. prausnitzii* (12, 38–42) in mediating diet-induced obesity and T2DM. A study by Everard et al. isolated *A. muciniphilia* (a mucin-degrading Gram-negative bacterium) within the mucous layer (30). Studies have demonstrated there to be a relative decrease in the population of *A. muciniphilia* in obese mice and mice with T2DM compared to lean cohorts (30). In addition, the use of prebiotics to reconstitute the population of *A. muciniphilia* resulted in improved metabolic profiles, reversal of high-fat diet-induced metabolic disorders (e.g., obesity, metabolic endotoxemia, adipocyte hypertrophy, and insulin resistance), increased intestinal levels of endocannabinoids that regulate inflammation, gut mucosal integrity, and intestinal peptide secretion, which ultimately results in restoration of the intestinal barrier (29). At present, the role of *A. muciniphilia* in the pathogenesis of obesity and T2DM are not known; however, studies suggest that the proximity of *A. muciniphilia* to the intestinal epithelium allows it to control gut barrier function, fat mass storage, and regulate glucose homeostasis (30).

*Faecalibacterium prausnitzii* is considered the prototypical anti-inflammatory bacterium and, thus, is among the most studied in a variety of disease states characterized and driven by inflammation (43, 44). Remely et al. recently studied the potential interaction between commensal microbiota in obese patients and patients with T2DM (38). These patients were compared to a lean cohort and evaluated over a 4-month intervention period (interventions comprised a GLP-1 agonist for those with T2DM and nutritional counseling for both groups). Through high-throughput sequencing and pyrosequencing, they found microbial diversity and the populous of *F. prausnitzii* to be significantly decreased in obese patients and patients with T2DM when compared to their lean counterparts. In addition, analysis of CpGs in the promoter region of FFAR3 demonstrated markedly lower methylation in obese patients and patients with T2DM, which increased in obese patients over the intervention period, thus showing a significant correlation between a higher body mass index and lower methylation of FFAR3. These results suggest that differing microbial makeups affect epigenetic regulation of FFAR-3 and possibly LINE-1 in obese patients and patients with T2DM.

## Targeted Therapy: Diet, Probiotics, Prebiotics, and Fecal Transplant

The composition and behavior of gut microbiota can be largely influenced *via* diet (45); therefore, the next logical question would be to see if diet has any contribution to the development or progression of diabetes. Since diabetes is known to be a multifactorial disorder, adjunctive therapeutic approaches beyond focused glycemic control may be helpful (46). Much of the research into this topic been focused on the use of prebiotics, probiotics, and fecal transplant.

### Prebiotics

Prebiotics are defined as non-digestible, fermentable carbohydrates (45, 47) that have the ability to influence the gut microbiota in order to provide a health benefit to the host (48); some examples of such prebiotics are inulin, oligofructose, and resistant starch (47). There are many natural foods that contain prebiotics in their raw form, such as chicory root, Jerusalem artichoke, barley, garlic, onion, globe artichoke, rye bran or grain, wheat bran, and asparagus; chocolate and white bread are examples of cooked or processed foods that contain prebiotics (45). A recent review of several randomized control trials of the effect of prebiotics on patients with prediabetes and T2DM by Barengolts et al. found that dietary changes (which include prebiotic supplementation) may ameliorate the effects of impaired insulin signaling and secretion (45).

In obese humans and genetically obese mice, a decrease in *Bacteroidetes* and an increase in *Firmicutes* phyla have been observed when compared to lean humans/mice (31, 45, 49, 50). Using genetically obese and diabetic mice, Everard et al. reported that a prebiotic-enriched diet resulted in changes of 102 distinct taxa, 16 of which displayed a >10-fold change in abundance. Of these results, two of particular importance was an increase in *Bacteroidetes* and a decrease in *Firmicutes* phyla; the obese mice fed a prebiotic-enriched diet showed a shift in two dominant phyla toward a profile more similar to lean mice (51). Prebiotics were also shown to improve many metabolic parameters, including lower fasting glycemic levels, improved glucose tolerance (52), reduced plasma triglyceride levels (47, 51), muscle lipid infiltration, adipose tissue mass, and oxidative stress, and increased leptin sensitivity (51).

Outside of affecting the composition of bacterial phyla, these fermentable carbohydrates play a role in the pathogenesis of T2DM and low-grade inflammation. One example is *Bifidobacteria*, whose growth can be nurtured *via* the supplementation of prebiotics, specifically dietary fructans. *Bifidobacteria* express the enzyme β-fructofuranosidase that allows them to break down fructans for energy (53). This is important because the number of *Bifidobacteria* is decreased in patients with diabetes mellitus, when compared to non-diabetics (53). In mice fed a high-fat diet supplemented with the prebiotic oligofructose, *Bifidobacterium* was shown to significantly and positively correlate with improved glucose tolerance, insulin secretion, and decreased inflammatory markers (54).

Prebiotics have also been shown to modulate the enteroendocrine and neuroendocrine brain systems *via* gastrointestinal peptides and neural signaling. Studies have shown that prebiotic supplementation can increase plasma glucagon-like peptide 1 (GLP-1) and plasma glucagon-like peptide 2 (GLP-2) (52, 54–56). In the setting of gut microbiota and diabetes, GLP-1 is thought to play a role in reducing appetite, fat mass, and hepatic insulin resistance (52), while GLP-2 is believed to reduce metabolic endotoxemia by decreasing intestinal wall permeability (57). Prebiotic treatment has also been shown decrease satiety and postprandial plasma glucose response after a standardized meal (54). It is possible that one of the mechanisms in which prebiotics contribute to changes in satiety and postprandial glucose excursion responses is through the modifications of these gastrointestinal peptides.

### Probiotics

Probiotics are living microorganisms that can improve the health of its host by altering the intestinal microflora when ingested (58). Research using *in vivo* and *in vitro* animal models has shown that probiotics may have a beneficial role in preventing and treating diabetes (46). *L. casei* is one example that has been proven to have anti-hyperglycemic capabilities in a diabetic mouse model. One group of researchers showed that supplementation of heat-killed *L. casei* cells exhibited decreased plasma glucose levels in a type 1 diabetic mouse model and prevented the onset of diabetes in a type 2 diabetic mouse model (26, 28). Another example included feeding *Lactobacillus rhamnosus* subtype GG to diabetic rats, which in 9 weeks had lowered the blood hemoglobin A1c level and improved glucose tolerance when compared to rats fed with normal diet (59). Along with improving diabetic parameters, probiotic supplementation in animals has also shown to decrease beta cell destruction, reduce oxidative damage to the pancreas, exhibit anti-inflammatory properties by increasing liver natural killer cells, reduce bacterial translocation from the intestine into the host, and decrease expression of pro-inflammatory cytokines (27, 60–62).

Unfortunately, there are few studies using probiotic therapy in diabetic human populations. Of the research available, it has been shown that probiotic supplementation can significantly reduce both the oxidative stress and associated inflammatory response, along with decreasing intestinal permeability (63). These favorable effects are thought to lead to greater insulin sensitivity, a decrease in autoimmune response (63), and an improvement in autonomic balance (see Figure 1).

A study in 2012 found that probiotic yogurt (already containing *Lactobacillus bulgaricus* and *Streptococcus thermophiles*) enriched with *Lactobacillus acidophilus* (La5) and *Bifidobacterium lactis* (Bb12) significantly decreased fasting blood glucose and hemoglobin A1c (58). Additionally, there was associated improved antioxidant status (increased erythrocyte superoxide dismutase, glutathione peroxidase activity) in patients with T2DM when compared to patients supplemented with regular, unenriched yogurt (containing only *L. bulgaricus* and *S. thermophiles*). The authors concluded that the decrease in oxidative stress likely resulted from anti-inflammatory and immunomodulatory properties of enriched probiotics. In that, oxidative/nitrosative stress plays a role in the initiation and progression of diabetes (64) and autonomic neuropathy (65); the addition of probiotics may be a potential adjunctive therapy in diabetics or perhaps a primary approach in pre-diabetics and in particular those with newly diagnosed neuropathy (66) and early neuropathy (65).

Cardiovascular disease is responsible for up to 65% of all deaths in diabetic patients (67). Probiotic supplementation can help improve the lipid profile of patients with T2DM. The same group from the study mentioned above showed that consuming probiotic yogurt enriched with *L. acidophilus* (La5) and *B. lactis* (Bb12) resulted in a 4.54% decrease in total cholesterol and a 7.45% decrease in LDL-C compared with patients who consumed regular, unenriched yogurt (58). There was no significant change in triglyceride or high-density lipoprotein cholesterol levels. Using the same probiotics, a different group of researchers found that supplementation with *L. acidophilus* (La5) and *B. lactis* (Bb12) caused a significant increase in HDL-C and a decrease in the ratio of LDL-C:HDL-C (68). Although these effects are mild compared with statins, the greatest predictor of cardiovascular events in diabetes is the loss of HRV combined with numb feet (6, 69). The effects of these probiotics on the ANS remain to be shown.

### Fecal Microbiota Transplant

Changes in the gut microbiota contribute to energy homeostasis and, therefore, the pathophysiological progression to diabetes and obesity. Studies have shown that alterations that occur within the gut flora allow obese animals to harvest a greater portion of energy from their diet (7). It has also been demonstrated that mice with sterile intestines that were fed a high-fat “western diet” did not develop obesity, insulin resistance, or dyslipidemia (70). Animal studies have shown that fecal microbiota transplant from a genetically bred obese mouse to a genetically bred lean mouse can transform the recipient to be the habitus of the donor (71).

Conceptually, a potential rebalancing of the gut flora in obese, diabetic patients could positively influence their energy homeostasis. A recent double-blinded, RCT studied the therapeutic effects of allogeneic lean donor feces infusion on insulin resistance in male patients with the metabolic syndrome (or, in the case of the control group, an autologous transplant) (72). Six weeks later, they found that the patients who had received an allogeneic transplant from lean donors had improved insulin sensitivity (median rate of glucose disappearance increased from 26.2 to 45.3 μmol/kg/min). They also found an increased abundance of butyrate-producing gut microbiota, which they concluded could be the reason for the improved insulin sensitivity, given the role of butyrate in increasing energy expenditure and mitochondrial function. Of note, butyrate stimulates the release of GLP-1 and enhances the beneficial effects of vagal activation. These favorable alterations in glucose metabolism support the notion that reconstituting commensal microflora with lean microflora might be a novel therapeutic intervention by reestablishing a “healthier” host environment.

## The Cholinergic Anti-Inflammatory Pathway in Diabetes and Obesity

The inflammatory response is controlled by neural circuitry of the autonomic nervous system. The afferent arc consists of nerves that sense the injury and infection, and this in turn activates a cholinergic anti-inflammatory pathway that modulates the response and is the potential target for future therapies of diabetes (73, 74). The lymphoid organs of the immune system are innervated by cholinergic, catecholaminergic, dopaminergic, and peptidergic neurons, and the neurotransmitters can interact with immune cells and alter their level of function. For instance, in the non-obese diabetic (NOD) mouse (an animal model for type 1 diabetes), neurons surrounding the pancreatic beta cells are lost before there is damage to the islet, and the loss of tonic inhibitory signals contributes to the subsequent beta cell destruction (75). In addition, destruction of the capsaicin-sensitive nerve fibers of the pancreas protect the beta cell from streptozotocin-induced beta cell injury (capsaicin and STZ) (76). Thus, it seems that to begin to understand the role of the autonomic nervous system in its more complex role in inflammation and autoimmunity, one needs to delve further into this complexity. Like St. Thomas, we need to probe deeper!

Watkins and colleagues discovered that sensory neurons detect the presence of inflammation in tissues (77). These responses to IL-1-induced inflammation were mediated by the vagus nerve and could be abolished by vagotomy or a selective competitive antagonist of the IL-1 receptor (78, 79).

There are a number of ligands other than IL-1 derived from macrophages, monocytes, and dendritic cells referred to as pathogen-associated molecular patterns (PAMPs), which activate TLRs, leading to increased expression of NF-κB and increased release of inflammatory cytokines such as TNFα and IL-6. Endogenous molecular products are also released from damaged cells and are referred to as damage-associated molecular patterns (DAMPs). Thus, the nervous system is capable of initiating a response to tissue injury and inflammation and can *per se* initiate a pro-inflammatory response.

The neurotransmitter of the efferent arc acetylcholine interacts with the innate immune cells that express the nicotinic acetylcholine receptor subunit α7 (α7nAChR). The receptor is widely expressed in neurons that function as ligand-gated ion channels encoded by CHRNA 7 on chromosome 15q14 and is a product of 10 exons yielding a mature protein of 50 kDa. α7nAChR has a tonic inhibitory role in the immune cells similar to the effects of the parasympathetic system on inhibition of heart rate. Exaggerated responses to inflammatory molecules are caused by vagotomy, whereas stimulation of the vagus downregulates the production of TNFα, IL-1, IL-6, and IL-8 but does not alter the anti-inflammatory cytokine IL-10 and TGFβ. This ACh activates the JAK/STAT pathway, affecting the inflammatory responses mediated by NF-κB and initiating the release of a variety of inflammatory cytokines. Thus, this is a defensive reflex protecting the organism from organ damage and death when exposed to syndromes of excess cytokine release such as infection, trauma, and stress.

We have hypothesized that in the metabolic syndrome and diabetes, there is a constant increase in low-grade inflammation mediated by a large cadre of exogenous and endogenous ligands (Figure 1). These, in turn, are capable of binding to the advanced glycation end product receptor (RAGE), thereby activating NF-κB pathway and increasing the production of inflammatory cytokines. In addition, there are a number of other ligands capable of activating the inflammatory cascade. DAMPs, for example, can stimulate innate immune responses culminating in NF-κB activation as with the ligands for RAGE. This is further compounded by the ligands binding to the TLRs, which potentiate the activation of NF-κB. The balance occurs by binding of acetylcholine to the α7nACHR receptor restraining the activation of NF-κB-mediated inflammation. Thus, the loss of autonomic control with reduction of parasympathetic activity, hallmark autonomic dysfunction in diabetes, initiates a cascade of inflammatory responses that if continued unabated will culminate in considerable morbidity and mortality. The possible approaches to enhancing parasympathetic function will be discussed below.

We (60) examined patients with newly diagnosed diabetes, established diabetes and healthy controls and analyzed a cadre of inflammatory markers in addition to time and frequency domain measures of autonomic function. Of great interest to us is the appearance before the advent of inflammation of loss of sympathetic/parasympathetic (S/P) balance. Early in the development of autonomic dysfunction, there is loss of S/P balance, and this correlates with an increase in circulating markers of inflammation, such as CRP and IL-6, and a reduction of the high molecular weight adiponectin/leptin ratios, which correlate with loss of parasympathetic function reflected by changes in the LF/a/HFa frequency ratios and a reduction in the RMSSD and SDNN in time and frequency evaluation of cardiac autonomic function (66, 80, 81). Activation of the efferent arm of the reflex arc (through the administration of an acetylcholine receptor agonist) causes a decrease in pro-inflammatory cytokine production and a reduction in disease severity (82, 83). In his review, Tracey points out a number of important clinical studies that show correlations between vagal nerve activity and inflammatory human diseases, such as rheumatoid arthritis and lupus (74). They considered that inhibition of macrophage function is mediated by Ach released by the vagus acting on specific alpha7nicotinic receptors expressed by the immune cell. Our results are in keeping with this research and suggest that such a reflex arc may be involved in the inflammation seen early in T2D. However, our studies implicate the hypothalamus as the conductor of the endocrine orchestra and show that the earliest changes that are detectable in the evolution of diabetes are abnormalities in autonomic balance. It is not beyond the realms of reason that we could reverse the unfortunate evolutionary profile by targeting the hypothalamic set point of autonomic balance, thereby restoring the efferent arm of the cholinergic anti-inflammatory pathway.

The relationship between binding of ligands to the pattern recognition AGE receptor (RAGE) and inflammation, gene expression, oxidative and nitrosative stress, and damage to the macro- and microvasculature is not entirely clear. Elevated levels of glucose bind to proteins and form AGEs, which bind to RAGEs. RAGE signaling activates NADPH oxidase and production of reactive oxygen species (ROS). Increased ROS increases advanced oxidation protein products (AOPPs), more AGEs, and AGE-modification of oxidized LDLs (oxLDLs). Furthermore, increased ROS may deplete glutathione, thereby suppressing glyoxalase I activity, a mechanism favoring further AGE accumulation. AGEs, AOPPs, macrophage glycoprotein (MAC-1), and AGE-oxLDL ligands of RAGE sustain stimulation of RAGE, and these processes, together with increased ROS, activate key transcription factors, such as nuclear factor-κB (NF-κB) and Egr-1, which increase gene transcription factors and activate inflammatory mechanisms. Consequences include increased migration and activation of RAGE-expressing neutrophils, monocytes/macrophages, T-cells, and dendritic cells. This results in the release of the pro-inflammatory RAGE ligands S100/calgranulins and high-mobility group protein box-1 (HMGB1). In this inflammatory environment, further AGEs may be formed as well. *Via* interaction with RAGE, these ligands magnify activation of NF-κB, Agr-1, and other factors, thereby amplifying cellular stress and tissue damage leading to neurovascular dysfunction. Soluble RAGE (sRAGE) is formed from the cleavage of RAGE by disintegrins, such as ADAM 10, a metalloproteinase, and β- and γ-secretases. sRAGE or a spliced variant (esRAGE) compete for binding of ligands to RAGE, and a deficiency could theoretically initiate the sequence of events activating an inflammatory cascade with an increase in the expression of pro-inflammatory cytokines [E-selectin, endothelin-1 tissue factor, vascular endothelial growth factor, and other pro-inflammatory cytokines (interleukin-6 and tumor necrosis factor-α)] and damage to neurons, kidney, eye, the vasculature, and even bone. Increasing sRAGE or its administration could competitively reduce activation of the AGE/RAGE pathway and it consequences (5).

Vagal chemoreceptors could be activated directly by substances, such as short-chain fatty acids that can be transported across the epithelial barrier to the portal circulation (84), or by paracrine mediators, such as 5-HT, histamine, CCK, ATP, or glucagon-like peptides released by the various mucosal epithelial layer taste cells (85, 86). That vagal mucosal chemoreceptors might be involved in activation of the “microbiome–gut–brain axis” (85), which is substantiated by animal studies where beneficial bacteria were applied to the epithelium at known concentrations and vagal nerve activity was recorded. There is now strong evidence from animal studies that gut microorganisms can activate the vagus nerve and that such activation plays a critical role in mediating effects on the brain and, subsequently, behavior. The anxiogenic effect of orally administered subclinical doses of *Campylobacter jejuni* on mice was associated with a significant increase in c-Fos expression in neurons bilaterally in the vagal ganglia and activated visceral sensory nuclei in the brainstem. The areas of brainstem activation, the NTS and lateral parabrachial nucleus, participate in neural information processing that ultimately lead to autonomic neuroendocrine and behavioral responses (87). These findings suggest that the influence of the bacteria on autonomic neurotransmission is mediated centrally, likely through histaminergic nerves and the suprachiasmatic nucleus (88). In a pioneering study, intraduodenal injection of a *Lactobacillus johnsonii* strain increased gastric vagus massed multiunit firing within 15 min of application (88). Given what is known of the vagal anti-inflammatory reflex, it seems plausible that gut microbiota-induced modulation of vagal mediated “periphery to brain” signaling could translate into changes in efferent neural pathways controlling immune responses (89).

## Conclusion

Currently, the depth and breath of the gut microbiome and its implications on obesity, the pathogenesis of diabetes, and its role in the autonomic cholinergic anti-inflammatory pathway that influences health and disease remain poorly understood. The microbiota gut–brain axis is a complex, bidirectional communication, which we are currently in the initial stages of understanding. It appears as though there are strong implications that intestinal dysbiosis *via* regulation of SCFAs, other paracrine mediators, the critical role of the gut microbiota in endotoxemia, and the effects of LPS on neurons are associated with obesity, metabolic syndrome, and diabetes and its complications.

Associated with the intestinal dysbiosis, animal studies have demonstrated an associated chronic low-grade inflammatory state, which is likely to play a contributing, if not a *pivotal*, role in diabetic-associated disorders. Clearly, the onset of diabetes is heralded by impairment of HRV, dictated by disordered hypothalamic function, which may owe its origin to the gut dysbiosis.

These seminal data offer significant translational opportunities for further research. Clinical studies evaluating the therapeutic and preventive strategies to prevent or alter this dysbiosis are clearly needed and offer potential new pathways for disease management.

## Author Contributions

AV and DJ: manuscript preparation and revision and final approval of the version to be published. PP and VN: manuscript preparation and final approval of the version to be published.

## Conflict of Interest Statement

The authors declare that the research was conducted in the absence of any commercial or financial relationships that could be construed as a potential conflict of interest.
